# No laughing matter: subacute degeneration of the spinal cord due to nitrous oxide inhalation

**DOI:** 10.1007/s00415-018-8801-3

**Published:** 2018-03-03

**Authors:** Stephen Keddie, Ashok Adams, Andrew R. C. Kelso, Benjamin Turner, Klaus Schmierer, Sharmilee Gnanapavan, Andrea Malaspina, Gavin Giovannoni, Ian Basnett, Alastair J. Noyce

**Affiliations:** 1 The Royal London Hospital, Emergency Care and Acute Medicine Clinical Academic Group, Neuroscience, Barts Health NHS Trust, London, UK; 2 National Hospital for Neurology and Neurosurgery and Department of Molecular Neuroscience, MRC Centre for Neuromuscular Disease, UCL Institute of Neurology, Queen Square, London, UK; 3 Department of Molecular Neuroscience, UCL Institute of Neurology, Queen Square, London, UK; 4 Barts and The London School of Medicine, Neuroscience and Trauma, Blizard Institute, Queen Mary University of London, London, UK; 5 Barts and The London School of Medicine, Preventive Neurology Unit, Wolfson Institute of Preventive Medicine, Queen Mary University of London, Charterhouse Square, London, WC1N 1PJ UK

**Keywords:** Subacute degeneration of the spinal cord, Myelopathy, Hydroxocobalamin, Vitamin B_12_

## Abstract

**Background:**

Whilst the dangers of ‘legal highs’ have been widely publicised in the media, very few cases of the neurological syndrome associated with the inhalation of nitrous oxide (N_2_O) have been reported. Here we set out to raise awareness of subacute degeneration of the spinal cord arising from recreational N_2_O use so that formal surveillance programs and public health interventions can be designed.

**Methods:**

Case series documenting the clinical and investigational features of ten consecutive cases of subacute degeneration of the spinal cord presenting to a hospital with a tertiary neurosciences service in East London.

**Results:**

Sensory disturbance in the lower (± upper) limbs was the commonest presenting feature, along with gait abnormalities and sensory ataxia. MRI imaging of the spine showed the characteristic features of dorsal column hyperintensity on T_2_ weighted sequences. Serum B_12_ levels may be normal because subacute degeneration of the spinal cord in this situation is triggered by functional rather than absolute B_12_ deficiency.

**Discussion:**

A high index of suspicion is required to prompt appropriate investigation, make the diagnosis and commence treatment early. This is the largest reported series of patients with subacute degeneration of the spinal cord induced by recreational use of N_2_O. However, the number of patients admitted to hospital likely represents the ‘tip of the iceberg’, with many less severe presentations remaining undetected. After raising awareness, attention should focus on measuring the extent of the problem, the groups affected, and devising ways to prevent potentially long-term neurological damage.

## Introduction

Nitrous oxide (N_2_O or laughing gas) has been used in clinical practice as a dissociative anaesthetic for over 170 years, but its recreational use has become widespread, facilitated by legal over-the-counter availability. N_2_O abuse is rapidly rising throughout the USA and UK, and has become the seventh most commonly used recreational drug according to the Global Drug Survey 2016 [1].

Several case reports have documented the potential for N_2_O to cause damage to the nervous system through interference with vitamin B_12_ metabolism, leading to megaloblastic anaemia and subacute degeneration of the spinal cord [2–5] which itself can be irreversible [6]. Here, we report the largest series of patients with neurological complications from N_2_O abuse, in an effort to raise awareness and prompt appropriate surveillance, so that an adequate public health response may be designed and implemented.

## Methods

All adult patients presenting between 1st of November 2016 and 1st May 2017 to the Emergency Department of the Royal London Hospital, with a history of N_2_O use and symptoms suggestive of subacute degeneration of the spinal cord were included. Two patients had a pre-existing diagnosis of subacute degeneration of the spinal cord and re-presented during this period (cases 8 and 10). Blood tests were performed to rule out alternative causes of myelopathy in most patients, including virology, anti-nuclear antibodies, anti-neutrophil cytoplasmic antibodies, anti-cardiolipin antibodies, aquaporin 4 antibodies and paraneoplastic antibodies. Nutritional deficiencies of copper and folate were also routinely tested. Cerebrospinal fluid examination was performed in four patients. All patients were seen and examined by an attending consultant neurologist and imaging reported by a consultant neuroradiologist. Where serum vitamin B_12_ level was normal, serum methylmalonic acid (MMA) was also measured. Where possible, patients were followed up in the outpatient department. Due to difficulties contacting patients (change of address, GP or phone number), not all patients were available to provide consent, and therefore all cases have been fully anonymised.

## Results

### Demographics

There were approximately 150,000 attendances to the Emergency Department over the 6-month study period. Table 1 summarises the clinical features of the ten study cases included in this report. The median age of patients was 22 years (range 17–26), three were women and seven were men. Seven patients were of Bangladeshi descent, one Asian, one mixed White-Caribbean and one was White. Eight were current smokers, six drank alcohol more than twice per week, and three used other drugs recreationally (two used cocaine and one marijuana). On average, patients used N_2_O around two-three times per week, and the number of N_2_O canisters consumed ranged between 75 and 2000 per week.

**Table 1:** Clinical and investigational findings in 10 patients with N_2_O-induced subacute degeneration of the spinal cord

Case	Number of reported N_2_O canisters per week	UL sensory	LL sensory	UL distal motor power	LL distal motor power	UL reflexes	LL reflexes	Gait ataxia	Bladder/bowel	Other	Vitamin B_12_ level (ng/L)	MMA level (μmol/L)	MRI at presentation	Follow-up MRI*
1	100	PP/JPS/LT loss	Vib loss	IV	IV	+	+++ C	Y	Nil	Myoclonic jerks, Uhtoff’s	138	0.7	C2–7 dorsal column hyperintensity and enhancement	Persistent dorsal column hyperintensity, enhancement resolved
2	1000	JPS/Vib loss	Vib loss	V	V	++	++	Y	Nil	Lhermitte’s, PA	169	ND	C1–7 dorsal column hyperintensity, cord atrophy	ND
3	300	HA/PP loss	HA PP, Vib loss	V	V	+	+++ C	Y	Constipation	PA	196	2.36	C1–6 dorsal column hyperintensity	ND
4	192	JPS/Vib loss	JPS/Vib loss	IV	IV	(+)	(+)	N	Nil	PA	> 2000***	ND	C1–7 dorsal column hyperintensity	ND
5	500	Normal	PP/JPS loss	V	IV	0	0	Y	Nil	Nil	186	3.45	C2–6 dorsal column hyperintensity	ND
6	600	Normal	HA PP/JPS/Vib loss	IV	IV	++	+++	Y	Nil	Nil	109	110	ND	ND
7	75	JPS loss	PP/JPS/Vib/LT loss	V	V	++	++	Y	Nil	PA	321***	0.16	C3–5 dorsal column hyperintensity	Complete resolution
8**	180	Normal	LT loss	V	V	++	0	N	Nil	Nil	229	14	C2–6 dorsal column hyperintensity	Persistent dorsal column hyperintensity
9	700	Normal	HA PP/JPS/Vib loss	V	IV	++	0	Y	Nil	Nil	169	0.61	C1–7 dorsal column hyperintensity	ND
10**	1500-2000	PP/JPS/Vib loss	PP/JPS/Vib loss	V	V	+	+	Y	Nil	PA	226	7.8	C4–7 dorsal column hyperintensity	Complete resolution

Power assessments according to MRC power score. Reflexes 0 = absent, (+) with reinforcement, + = diminished, ++ = normal, +++ = brisk, C = with clonus, UL = upper limbs, LL = lower limbs. MMA = methylmalonic acid. B_12_ reference range 191-900 ng/L, MMA 0.0-0.29umol/L

*HA* hyperaesthesia, *LT* light touch sensation, *PP* pin prick sensation, *JPS* joint position sensation, *Vib* vibration sensation, *PA* pseudoathetosis. *ND* not done/not available

* Follow-up MRI was performed at 5 months for case 1, 8 months case 7, 16 months case 8 and 27 months case 10

** both patients re-presented following a previous diagnosis of subacute degeneration of the spinal cord

*** Already on B_12_ replacement

### Clinical features

Altered sensation in the limbs was the predominant presenting feature (seven had symptoms in the upper limbs and all ten had symptoms in the lower limbs). Strength was well preserved in most patients. Additional clinical features were gait ataxia (*n* = 8), falls (*n* = 3), Romberg’s sign (*n* = 6), pseudoathetosis (*n* = 5), Lhermitte’s phenomenon (*n* = 1), Uhtoff’s phenomenon (*n* = 1), and segmental myoclonus (*n* = 1).

### Investigations

Median haemoglobin level was 148 (range 117–170 g/L). All patients had normal mean cell volume (median 92.4; range 89.8–94.8 fL). Four patients had low vitamin B_12_ levels (median 191; range 109–2000 ng/L). MMA was measured in eight patients (median 2.9; range 0.16–110 μmol/L) and was not taken in patients whose B_12_ level was below normal or elevated as a result of replacement. MMA was elevated in seven of the eight patients. Four of these could be deemed clinically relevant/revealing, with an associated normal B_12_ level. Three were ‘complimentary’, with an associated low B_12_ level. The MMA level was normal in one patient whose vitamin B_12_ level was low (case 7), but it transpired that they had been aware of the risks of myelopathy and had been concurrently taking oral B_12_ 1000 μg once a day as prophylaxis. Despite this they had nonetheless developed subacute degeneration of the spinal cord.

A CSF examination was performed in four patients. White cell counts were < 1 in all cases. Protein was raised in two patients at 0.5 and 0.7 g/L (normal ≤ 0.4 g/L). Glucose was within normal limits in all. Unmatched oligoclonal bands were demonstrated in one patient, the significance of which is unclear but would suggest a degree of intrathecal immunological response.

**Fig. 1:**
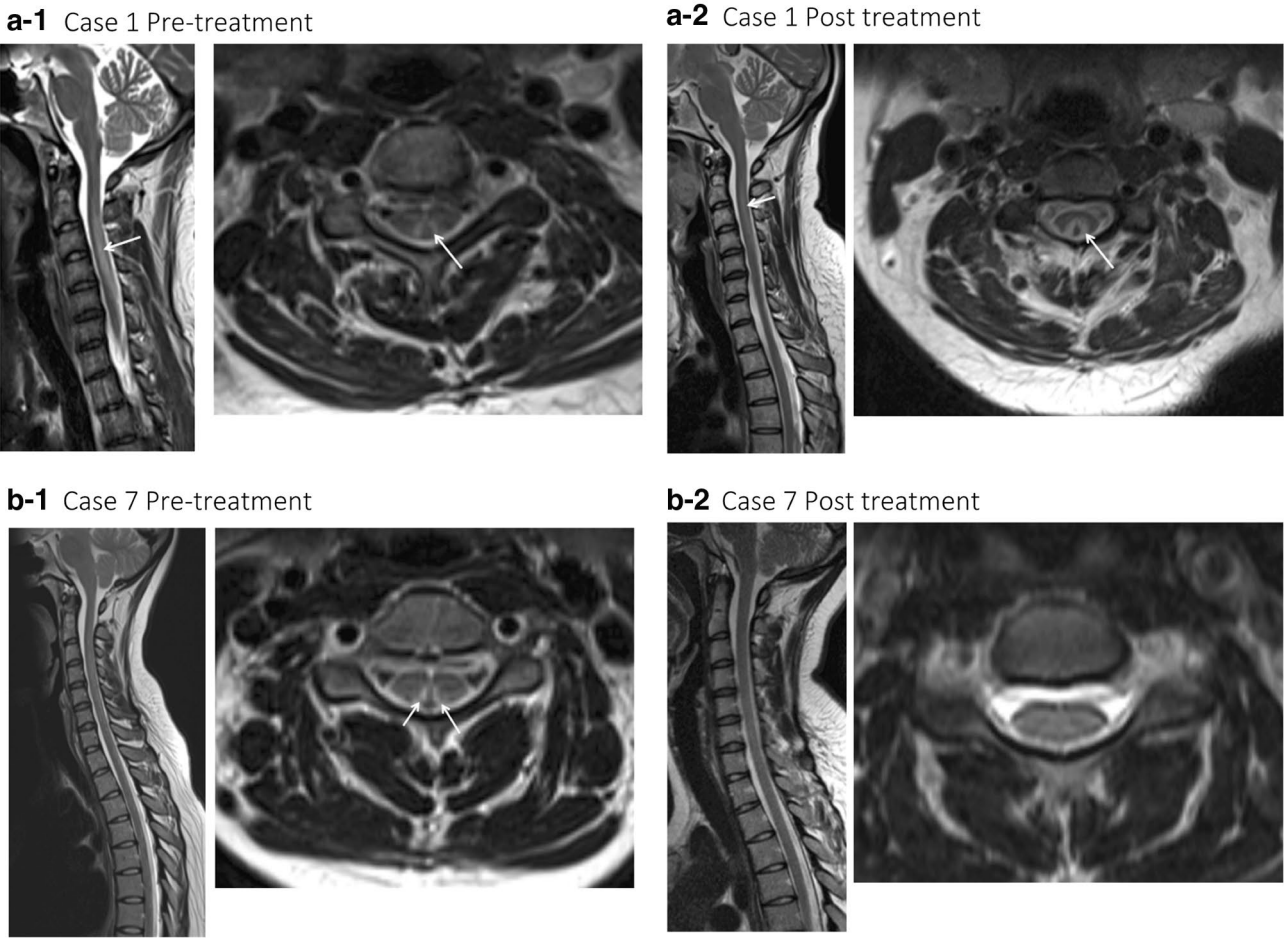
Pre- and post-treatment sagittal and axial T2 weighted MRI imaging of the cervical cord in N_2_O-induced subacute degeneration of the spinal cord. **a-1**: Sagittal T2 weighted sequence demonstrating extensive cord signal change from the C2 to the C7 level within the dorsal aspect of the cord confirmed on the axial sequences, with enhancement (not shown). **a-2**: Taken 5 months following showing persistent abnormal T2 signal change within the dorsal columns on this sagittal T2 weighted sequence; however, the abnormal enhancement had resolved (not shown). **b-1**: Sagittal T2 weighted sequence demonstrating cord signal change from C3 to C5 within the dorsal aspect of the cord confirmed on the axial sequences, without enhancement, and resolution over time (**b-2**)

MRI of the spinal cord was performed in nine patients and showed T_2_ signal change affecting the dorsal columns of the cervical spine, consistent with subacute degeneration of the spinal cord (see Fig. 1). In one patient, pathological enhancement was detected initially. Follow-up MRI was performed in four patients after an average of 14 months (range 5–27 months) from presentation. In two patients the signal change persisted and in the other two it had resolved. In the cases where MRI signal change had resolved (cases 7 and 10), both received treatment for a minimum of 4 months. Case 7 had abstained from N_2_0, had been taking oral B_12_ prior to presenting, and was asymptomatic following treatment. Case 10 continued to use N_2_0 once a fortnight and experienced persistent paraesthesia in the feet. The patient also had poor diabetic control, and nerve conduction studies demonstrated mixed axonal and demyelinating features consistent with diabetic neuropathy.

### Management

All patients received hydroxocobalamin injections, 1 mg intramuscularly on alternate days, for a median of 2 weeks. Four patients were lost to follow-up. Of the remaining six patients, two recovered without residual symptoms, three continued to have paraesthesia in the feet and one continued to have paraesthesia, gait ataxia, and proprioceptive sensory loss to the ankles (case 10 above).

## Discussion

**Fig. 2:**
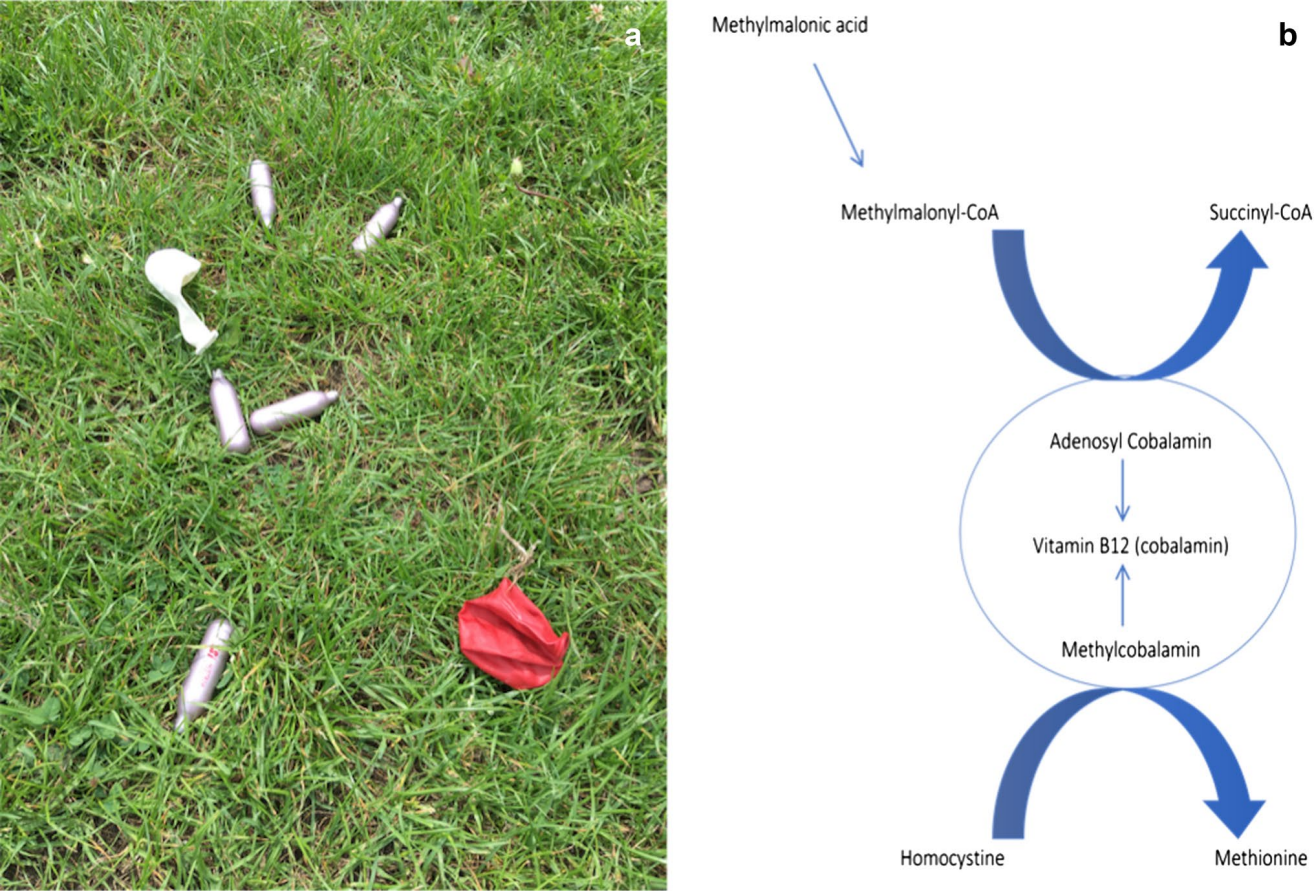
**a** Discarded nitrous oxide canisters and balloons in East London. **b** Metabolic pathway of vitamin B_12_ involved in pathogenesis of N_2_O-induced subacute degeneration of the spinal cord. Vitamin B12 is a cofactor in the conversion of methylmalonyl-CoA to succinyl-CoA and homocystine to methionine. Non-functioning B_12_ leads to accumulation of methylmalonic acid and homocystine, which can be tested in the patient sera when B_12_ levels appear normal, suggesting a ‘functional; B_12_ disorder. Figure created using Microsoft Word

Recreational use of N_2_O is largely through use of whipped cream chargers or ‘whippets’ bought from ‘head shops’ or online (see Fig. 2a). Whippets fit onto a dispenser, which is attached to a balloon that enables gas inhalation. Inhalation results in almost immediate psychotropic effects including euphoria, giggling, distortion of sound and mild hallucinations, peaking after about 20 s before rapidly diminishing. Users often feel entirely normal within 2 min following inhalation. Side effects of dissociation, blurred vision, acute ataxia, nausea and headache have been reported [7].

Recreational use of N_2_O in the UK has recently increased [7]. According to the Global Drug Survey, which collected data from over 100,000 drug users in 20 countries, N_2_O is the seventh most popular recreational drug worldwide, and the fourth most commonly used by UK nightclub attendees, with 48% admitting to using the drug [1]. Since the Psychoactive Substances Act came into effect in the UK in May 2016, it has become illegal to supply N_2_O for recreational consumption. Two recent high-profile cases of failed prosecutions for N_2_O supply have forced the Crown Prosecution Service to consider the implications for future cases [8]. Nonetheless, whippets are still being sold at a price of approximately £30 ($40) for 96 canisters.

Previous reports have been published of patients presenting to hospital with acute pain and administration of medicinal N_2_O excessively, with subsequent development of subacute degeneration of the spinal cord [9]. However, routine use in anaesthesia or dental procedures only carries a risk of subacute degeneration of the spinal cord in patients with low or low/normal serum vitamin B_12_ [2, 6].

The deleterious effects of N_2_O overuse are secondary to its interference with the action of vitamin B_12_. N_2_O causes oxidation of cobalt ions in vitamin B_12_, leading to its inactivation (see Fig. 2b). This results in reduced recycling of homocystine to methionine, preventing methylation of myelin proteins, thereby causing demyelination.

Identification of subacute degeneration of the spinal cord secondary to N_2_O abuse requires a high index of suspicion and a thorough history, supported by the clinical examination, laboratory data and radiological findings. A normal vitamin B_12_ level does not rule out the possibility of N_2_O-induced subacute degeneration of the spinal cord, given that functional B_12_ deficiency can occur in the presence of normal serum vitamin B_12_ levels. In such cases, detecting raised MMA and homocystine, which rely on normally functioning vitamin B_12_ for their metabolism, can give crucial clues to the diagnosis. Sagittal MRI imaging demonstrates a classic appearance of T2 hyperintensity in the dorsal spinal cord. Contrast enhancement is uncommon.

The management of patients includes educating them about the risks of N_2_O and vitamin B_12_ replacement using high-dose intramuscular hydroxocobalamin (1 mg on alternate days until no further neurological improvement, followed by 1 mg every 2 months) [10]. Neurological recovery may be incomplete, particularly when patients continue to use N_2_O [6].

Our series revealed that N_2_O-related subacute degeneration of the spinal cord tended to occur in young people. While most were smokers, unexpectedly, and not in keeping with the habits of other drug users [11], the majority did not drink alcohol or take other recreational drugs. However, this may also reflect the culture of the population local to our hospital. The finding that 70% of patients were of Bangladeshi origin emphasises the importance of considering recreational drug use even when religious and/or cultural assumptions may dissuade clinicians from this line of enquiry. The high Bangladeshi proportion also raises questions whether ethnicity-related metabolic, nutritional or genetic predispositions influence functional B_12_ metabolism and predispose to neurological damage. Anecdotally, many N_2_O users were not aware of the harmful effects of the drug and believed that because it was not previously illegal to consume, it was also safe to use.

The true scale of the problem is hard to gauge, but may only be improved through collaborative working by health professionals in neurology, public health and emergency medicine. Better recognition and accurate coding at the point of access to care will help ascertain the incidence and prevalence of neurological damage related to N_2_O, and plan an effective public health response.
